# Evaluating teaching quality in colleges using combination of artificial neural networks (ANNs) and black hole optimization (BHO)

**DOI:** 10.1016/j.heliyon.2023.e20687

**Published:** 2023-10-05

**Authors:** Li Guan

**Affiliations:** School of Marxism, Dalian Polytechnic University, Dalian, 116034, China

**Keywords:** Quality assessment of education, English language teaching, Artificial neural network, Black hole optimization, Ensemble learning

## Abstract

Automated evaluation of educational quality can be an effective measure of the efficiency of teaching methods, enabling the determination of the best teaching approach based on the educational environment conditions. Machine learning techniques have been used in various research studies as tools for automated evaluation of teaching quality. However, the low accuracy of classical learning models in quantitative quality estimation remains a challenge. To address this issue, one can utilize optimization strategies for learning models or combine multiple learning models as an ensemble system. These aspects are studied in the current paper. In the proposed method, automated evaluation of English teaching quality is performed through two phases: "identification of prominent quality evaluation indicators" and "quality estimation based on ensemble learning." In the first phase, the Black Hole Optimization (BHO) algorithm is used to determine the minimum number of indicators required for accurate quality estimation. In the second phase, the proposed method combines multiple artificial neural networks (ANNs) to predict education quality. In this ensemble model, the configuration and weight vector of each ANN are adjusted by the BHO algorithm to minimize training error. Then, an averaging strategy is employed to determine the final output of the ensemble system. The performance of the proposed method is evaluated using real English teaching data. Based on the results, the proposed method can achieve an average accuracy of 98.53 % in estimating English teaching quality, which represents a 2.19 % improvement compared to previous methods.

## 1 Introduction

English is one of the most widely spoken languages in the world and English language skills are considered as one of the criteria for evaluating the level of literacy in most societies [1]. For this reason, English teaching is considered as one of the main subjects in different levels of education [2]. Among them, teaching English at higher levels is of particular importance because learning English can be beneficial for college students in terms of cognitive development, accessing new practical resources, improving quality of life, and increasing job opportunities [3]. Providing these opportunities for college students can only be possible through proper English language instruction [4], which requires the use of effective and high-yielding teaching methods [5,6]. Determining a suitable approach for English language instruction is a challenging process and should take into account parameters such as cultural characteristics, students' native language, and the structural features of the educational system [7,8]. Based on this, it is not possible to consider a single teaching method effective in different circumstances, and the quality assessment of English language teaching methods should be carried out continuously.

Currently, in most educational systems, the quality assessment of education is carried out through the supervision of experts and reviewing the grades obtained from exams using a traditional approach, which is time-consuming, costly, and prone to errors [9]. Therefore, in recent years, efforts have been made to automate the process of quality assessment. Machine learning techniques, which are highly flexible, have been utilized in various research studies. However, the low accuracy of classical machine learning models in quantitatively estimating the quality of teaching remains a challenge.

The main question that the attempt to answer has motivated the current research, is how to improve the performance of machine learning techniques in the problem of evaluating the quality of English teaching. Answering this main question will be possible by addressing several sub-issues, which include: how to determine the most relevant factors with the quality of English teaching and also, how to improve the accuracy of machine learning models based on the problem conditions. The objectives of the current research are to answer these questions, and to achieve them, a combination of evolutionary algorithm and ensemble learning has been used.

To address the main research question, there are three feasible solutions: the first solution is to determine appropriate and relevant indicators of teaching quality, which should be selected correctly. In addition to improving accuracy (through eliminating irrelevant data), this operation can also improve the processing speed of the algorithm (through reducing the dimensionality of data). Therefore, determining the relevant indicators of teaching quality was the first method used in the current research. The second solution to enhance accuracy is to optimize the configuration of learning models and adjust their parameters. This process can be conducted empirically or through evolutionary search methods. Empirical model configuration has been used in some previous research studies, but it is time-consuming and inefficient when the parameter space is extensive. On the other hand, evolutionary search methods can perform this task automatically and adaptively. This, has caused the evolutionary search methods to be considered as a suitable solution to solve this problem in the current research. The third solution is to combine multiple learning models within ensemble systems. It has been theoretically proven that ensemble systems can effectively improve the accuracy of machine learning models [10]. This feature made the ensemble technique to be chosen as one of the appropriate tools to fulfill the research objectives. In this article, these three solutions are examined based on evolutionary algorithms and machine learning techniques. The proposed method not only determines the most relevant factors for the quality of English teaching, but also utilizes an ensemble system based on ANNs to estimate the quality of teaching. In this system, the parameters of each learning model are adjusted using optimization techniques. The contribution of this is twofold.

• In the current research, the most relevant indicators related to the quality of English teaching have been identified using optimization techniques. To achieve this, a set of candidate indicators related to English teaching is introduced, and the BHO algorithm and correlation measure are utilized to determine prominent indicators in quality assessment.
• In this article, a novel estimation model based on the ensemble of ANNs is proposed for evaluating the quality of English teaching. This model consists of three ANNs whose parameters, including the number of neurons in the hidden layer and weight vectors, are optimized through the BHO algorithm. The model utilizes an averaging strategy to estimate the teaching quality. Also, the efficiency of combining these three ANNs has been evaluated (in section 4) and the effect of their cooperation in increasing the accuracy of the system has been studied.

The structure of the current article is as follows: Section 2 includes a review of previous similar studies. Section 3 describes the details of the proposed method for evaluating the English teaching quality. Section 4 presents the research findings and discusses the results. Section 5 concludes the article.

## 2 Related works

In [11], an algorithm for evaluating the quality of education using Markov chain is proposed. This research provides a quantitative evaluation method where the importance of different educational indicators is determined through a Markov chain model. It also utilizes a weighting model for ranking quality indicators. These two categories of indicators are combined and the weight factors of education are calculated through the maximum eigenvector to estimate the quantitative quality of education. In Ref. [12], an online system for evaluating the quality of English language education based on Support Vector Machine (SVM) and complex networks is presented. In this method, the quality-related indicators of English language education are preprocessed, and Principal Component Analysis (PCA) is used to reduce the dimensionality of the data features. By reducing the dimensionality, the complexity of the problem can be reduced, and the quality evaluation can be performed by models with simpler structures. Thus, a combination of SVM and complex networks is used for evaluating the quality of education.

In [13], data mining techniques and the sixth-generation network-based Internet of Things (IoT) platform are used for monitoring and evaluating the quality of education. In this model, the teaching processes (including teaching methods, classroom management, and learning supervision) and student learning (including overall and partial assessments, opinions, and suggestions) are continuously monitored through IoT devices. The collected information from these processes is analyzed using association rule techniques to assess the quality of education. The feedback analysis strategy is also employed in this model for continuous improvement of education quality. In Ref. [14], wireless sensor network communication platforms and data mining techniques are utilized for evaluating the quality of education. In this study, firstly, a wireless sensor network-based communication model is proposed to monitor the processes related to education quality in classrooms. Then, a fuzzy comprehensive evaluation method is presented to assess the quality of education using the collected information. Finally, convolutional neural networks (CNNs) are used for teaching quality detection. This strategy is suitable for implementation in virtual learning environments.

In [15], a method for evaluating the quality of English language education in colleges based on the COmbinative Distance-based ASsessment (CODAS) method is presented. This research introduces a group decision-making approach based on multiple criteria, where the basic CODAS approach is combined with the Interval-Valued Intuitionistic Fuzzy Sets (IVIFS) strategy. In the IVIF-CODAS method, the weights of English language education quality indicators are determined objectively using the CRiteria Importance Through Intercriteria Correlation (CRITIC) method. Additionally, a new distance measure for intuitionistic fuzzy sets with interval-valued memberships is introduced to estimate the quality of education in IVIF-CODAS. In Ref. [16], a method for evaluating the quality of education based on fuzzy sets with weighted interval-valued dual hesitant fuzzy sets (WIVDHFS) is employed. The use of the weighted IVDHFS strategy can lead to a more detailed description of the importance of the relationships between indicators and education quality, thereby increasing the accuracy of the evaluation. Then, using t-norm and t-conorm Archimedean operators, a set of basic rules for quality evaluation based on the combination of WIVDHFS is provided, generating the output of a weighted fuzzy model.

In [17], a new method for evaluating the quality of education is presented, which utilizes 18 indicators categorized into four groups. The DANP (Decision-making Analytic Network Process) method is employed to examine the effectiveness of these indicators on education quality. The research demonstrates that the most important indicators affecting the estimation of education quality are, in order of importance: training performance, training process, learning environment, and input resources. Among them, learning environment and input resources are causal indicators, while training performance and training process are effect indicators. In Ref. [18], a method for evaluating the quality of education in large virtual classes is proposed. In this study, a macro-data statistical analysis model is used to identify fuzzy parameters and describe the indicators. The combination of macro-data analysis and clustering methods is then employed to describe a qualitative indicator. Finally, a decision tree model is utilized to estimate the education quality.

In [19], a machine learning-based method for evaluating the quality of English language education is proposed. In this method, initially, 10 quality indicators are introduced in four categories: class performance, instructional tools, teaching behavior, and curriculum. The PCA technique is then employed to determine the role of indicators in evaluating education quality through the analysis of obtained eigenvectors. Finally, machine learning techniques and fuzzy evaluation are utilized to predict the quality of English language education based on relevant indicators. In Ref. [20], data mining techniques are employed to evaluate the performance of faculty members in colleges. Three categories of indicators related to the quality of teaching are considered: teaching behavior, teaching method, and readiness for teaching. Then, the Classification and Regression Tree (CART) algorithm is used to rank these indicators and predict the education quality based on them.

In [21], a neural network-based method for evaluating the quality of practical agricultural education in technical and vocational schools is presented. In this approach, data related to the interaction between instructors and students collected through computers or mobile phones, and input indicators are created based on this information. Finally, a backpropagation neural network model is used to estimate the education quality. In Ref. [22], a method for identifying prominent indicators in evaluating education quality in colleges is presented. Four sets of indicators are examined in this study: curriculum, teaching methods, assessment, and student communication. Both inferential statistical analysis (analysis of variance) and descriptive analysis (mean, rate, and frequency) are used to evaluate the relationships between indicators and education quality. The results indicate that indicators related to the curriculum are of the highest importance and have the strongest correlation with education quality in colleges.

Research in Ref. [23], has used the Gaussian process for evaluating English teaching quality. This model, utilized Gaussian mixture model for exploring the distribution features of data. Also, a Relevance Vector Machine (RVM) was used for evaluation. In Ref. [24], a Radial Basis Function (RBF) ANN was introduced for evaluating the quality of English teaching in colleges. This ANN has a static structure with 31 neurons in its hidden layer which has been trained using conventional RBFNN training algorithm. In Ref. [25], a big data-based model for evaluating the quality of English teaching in colleges was presented. This research uses PCA for reducing the dimensionality of data. Using feature extraction methods such as PCA, is effective in improving the computational efficiency; however, this technique is not effective in determining the importance of indicators and removing the irrelevant ones. After reducing the dimensionality of data, the SVM classifier of used for evaluating English teaching quality.

## 3 Proposed method

The evaluation of quality can be done in the form of a quantitative variable (e.g., a rating from 1 to 5) or a qualitative variable (e.g., good or bad). Assessing the quality of education based on quantitative criteria is more complex and poses greater challenges. The proposed method in this paper attempts to reduce this complexity by decomposing the problem into a set of smaller sub-problems. Accordingly, the proposed solution for quantitative evaluation of the English teaching quality is performed through the following steps.

1. Selection of prominent indicators for evaluating the quality of teaching.
2. Estimation of teaching quality based on the ensemble of ANNs.

**Fig. 1:**
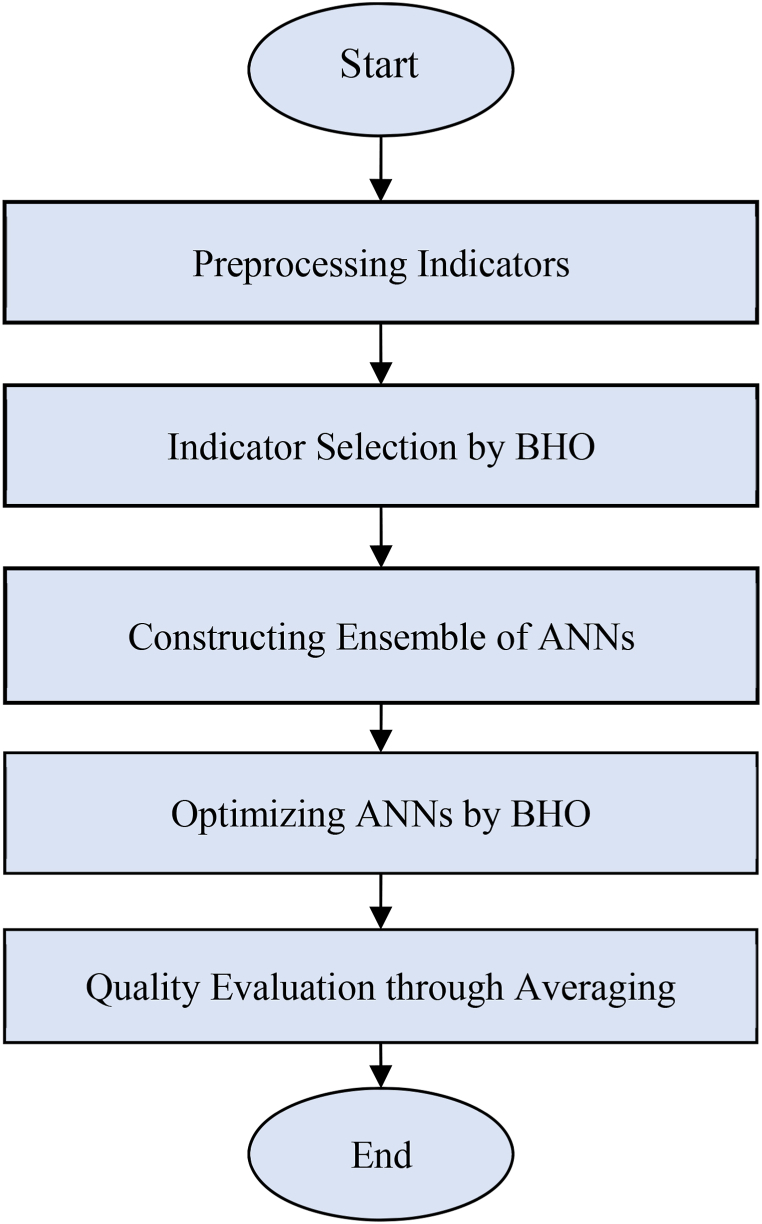
Flowchart of the proposed method.

The objective of the first step in the proposed method is to determine an optimal subset from candidate indicators for evaluating the English teaching quality, with the aim of maximizing the estimation accuracy. To achieve this, the BHO algorithm is employed. This optimization algorithm analyzes the correlations between indicators to identify the optimal set for maximizing the estimation accuracy. Once this subset is determined, in the second step of the proposed method, an ensemble model based on the combination of ANNs is utilized to estimate the English teaching quality. Since no stable computational model exists to guarantee the maximum efficiency of ANNs, the black hole optimization algorithm is also employed in this step to determine the optimal configuration of each learning model. In this step, the optimization algorithm strives to determine the number of hidden neurons and the weight vector of the neural network in a way that minimizes the estimation error of each model. Finally, the averaging strategy is employed to combine the estimated results of optimized neural networks in the proposed ensemble system. The combination of outputs, based on calculating the average, leads to a reduction in the final error of the ensemble system. The flowchart of the steps in the proposed method is depicted in Fig. 1.

### 3.1 selection of prominent indicators for evaluating the teaching quality

Appropriate indicators for accurately assessing the English teaching quality should possess features such as high correlation with quantitative quality characteristics, suitable describability, and ease of collection. The high correlation with quality characteristics means that an indicator is suitable if it exhibits a pattern similar to the level of quality. On the other hand, suitable describability of an indicator implies that it can be assigned a precise and processable value (nominal or numerical). The first step in the proposed method focuses on determining a set of suitable indicators for evaluating the English teaching quality. For this purpose, an initial set of candidate indicators is introduced, and an optimization strategy is employed to identify the prominent indicators. In the process of collecting candidate indicators, an effort has been made to consider various potential factors as well as different spaces of English language education (online or in-person training). The set of candidate indicators considered for evaluating the English teaching quality is presented in Table 1. The introduced set of candidate indicators is classified into five main categories: class performance, teaching tools, teaching environment, teaching behavior, and teaching plan. Each of these categories includes a set of specific indicators identified by an identifier such as $I_{x,y}$, where x represents the major indicator identifier and y represents the minor indicator identifier.

**Table 1:** Candidate indicators for evaluating the quality of English language teaching.

Major Indicator	Minor indicator	ID
Teaching Plan	Proper and Scientific Instruction	$I_{1,1}$
	Timely Presentation of Materials in Sessions	$I_{1,2}$
	Prioritizing Topics and Adhering to the Syllabus	$I_{1,3}$
	Referring to the Curriculum in Classes	$I_{1,4}$
Teaching Behavior	Ability to Maintain Order	$I_{2,1}$
	Mastery in Presenting Materials	$I_{2,2}$
	Responding to Questions Raised in Sessions	$I_{2,3}$
	Rate of English Language Use During Instruction	$I_{2,4}$
	Avoidance of Tangential Issues During Instruction	$I_{2,5}$
	Sustained Interaction Between Instructor and Learner	$I_{2,6}$
Teaching Environment	Continued Interaction Outside the Classroom Through Online Tools	$I_{3,1}$
	Use of Instructional Aids to Enhance Teaching	$I_{3,2}$
	Instructor's Attention to (LSRW) Skills in Class	$I_{3,3}$
	Standard Deviation of Students' Ages in the Class	$I_{3,4}$
	Male-to-Female Ratio Among Learners	$I_{3,5}$
	Availability of a Platform for Questions and Answers After Class	$I_{3,6}$
Teaching Tools	Precise Instructor Responses to Questions Raised in Class	$I_{4,1}$
	Teaching Based on Concrete Examples and Understandable Scenarios	$I_{4,2}$
	Efforts to Improve Learners' LSRW Skills	$I_{4,3}$
	Teaching Based on Surface-to-Deep Level Strategies	$I_{4,4}$
Class Performance	Learner Participation Rate in Answering During Instruction	$I_{5,1}$
	Response Rate to Questions by Learners in Sessions	$I_{5,2}$
	Rate of English-to-Native Language Conversations by Learners	$I_{5,3}$
	Learner Participation Rate in Instructional Discussions	$I_{5,4}$

In the set of indicators mentioned in Table 1, all the minor indicators related to the teaching plan are nominal. In the set of indicators related to teaching behavior, only indicator I_2.4_ is described numerically, while the other indicators are nominal. In the set of educational environment indicators, two indicators, I_3.4_ and I_3.5_, are numerical, and the other indicators are nominal. All the minor indicators of the teaching tools are nominal. Finally, all the indicators in the class performance set are numerical. It should be noted that the nominal indicators are assigned the values "Yes" or "No," while the numerical indicators (except for the two indicators I_3.4_ and I_3.5_, which have no upper bound) are assigned real numbers in the range [0, 1].

To select prominent indicators from the proposed candidate set, all the nominal indicators are first converted to numerical values. For this purpose, all the "No" values are replaced with zero, and the "Yes" values are replaced with one. By doing this, all the indicators will be described numerically. Then, the BHO algorithm is used to select an optimal subset of these indicators. BHO is a population-based algorithm whose features such as the simplicity of the mechanism and effective search of the problem space make it possible to use it in solving various problems. In this algorithm, each candidate solution is considered as a star and the best solution is modeled as a black hole. This algorithm, simulates the movement pattern of stars and the behavior of black holes in swallowing stars around them. This mechanism can effectively prevent candidate solutions from being trapped in local optima. These characteristics have made it possible to consider BHO as an efficient tool for solving the feature selection problem in this research. To do this, the problem of selecting prominent indicators is modeled as an optimization problem.

In the optimization model used in the proposed method, the selection state of each candidate indicator is considered as an optimization variable. Therefore, the number of optimization variables in the BHO algorithm will be equal to the number of candidate indicators, where each variable can have a value of zero (indicating non-selection of the indicator) or one (indicating the selection of the indicator). Based on this, each solution vector in the BHO algorithm is represented as a binary vector of length 24, where each indicator vector can be a potential solution for the selection of prominent indicators in evaluating the English teaching quality.

In the proposed optimization model, the selection of indicators is based on correlation criteria. In this case, the objective is to select the minimum number of indicators that have the highest correlation with the English teaching quality. This objective can be formulated as follows:

(1) $$Objective_{1}\left(\vec{x}\right)=\frac{1}{K}\sum_{k=1}^{K}\left|Corr\left({\vec{x}}_{k}.Q\right)\right|$$

Where, K determines the number of selected indicators in the solution vector, which can be extracted by calculating the sum of values of "1″ in the solution vector. The variable $x_{k}$ describes the values of the k-th selected indicator in the solution vector x. The function Corr(X.Y) indicates the correlation value between the values of two indicators, X and Y. Finally, Q represents the quantitative vector of education quality, which is considered as the objective variable. In fact, equation (1) calculates the average correlation between selected indicators and the target variable.

On the other hand, the selected set of indicators should not contain redundant information. To meet this requirement, the correlations between the selected indicators are calculated, and efforts are made to minimize this value. This objective can be formulated as follows:

(2) $$Objective_{2}\left(\vec{x}\right)=\frac{1}{K^{2}}\sum_{i=1}^{K}\sum_{j=1}^{K}\left|Corr\left({\vec{x}}_{i}.{\vec{x}}_{j}\right)\right|$$

Equation (2), calculates the average correlation between each pair of selected indicators. The proposed approach attempts to minimize the above objective. Considering objective 1 (which should be maximized) and objective 2 (which should be minimized), the fitness of each solution vector in the proposed optimization model can be calculated by combining the mentioned objectives, as follows:

(3) $$Fitness\left(\vec{x}\right)=\frac{\frac{1}{K^{2}}\sum_{i=1}^{K}\sum_{j=1}^{K}\left|Corr\left({\vec{x}}_{i},{\vec{x}}_{j}\right)\right|}{\sqrt{K}\left(1+\frac{1}{K}\sum_{k=1}^{K}\left|Corr\left({\vec{x}}_{k},Q\right)\right|\right)}$$

The numerator part of the equation above aims to identify indicators with minimum correlation with each other, while the denominator part evaluates the correlation between the selected indicators and the objective variable. Additionally, it aims to minimize the number of selected indicators (by considering factor $\sqrt{K}$ which is equivalent with minimizing selected indicators). Based on the proposed model, the selection of prominent indicators for evaluating the English teaching quality is performed by the BHO algorithm through the following steps:

Step 1 Randomly generate an initial population of solution vectors in the form of binary strings.

Step 2 Calculate the fitness of each solution vector based on equation (3).

Step 3 Determine the solution with the minimum fitness as the black hole $X_{BH}$.

Step 4 Shift the position of each solution vector, such as $X_{i}$, according to Ref. [26].

(4) $$X_{i}=X_{i}+rand.\left(X_{BH}-X_{i}\right)$$

In equation (4), the vector $X_{BH}$ represents the position of the black hole, and the vector $X_{i}$ represents the position of star/solution i in the problem space. Additionally, rand denotes a random number in the range (0, 1).

Step 5 Calculate the threshold distance for a star to be swallowed by the black hole as [26]:

(5) $$R=\frac{fitness\left(BH\right)}{\sum_{i=1}^{N}fitness\left(i\right)}$$

In equation (5), N represents the number of solution vectors in the BHO algorithm.

Step 6 If the fitness of a solution vector is lower than the black hole's fitness, replace it with the current black hole.

Step 7 Remove solution vectors that are closer to the black hole than the threshold distance R and replace them with a new random solution vector.

Step 8 If the number of algorithm iterations reaches the threshold T, proceed to the next step. Otherwise, repeat the search from step 2.

Step 9 Return the black hole with the lowest fitness as the best discovered solution.

After determining the optimal solution based on the above steps, the selected indices are extracted from this solution, and the input for the proposed ensemble model takes shape in the second step.

### 3.2 quality estimation based on the ensemble of ANNs

**Fig. 2:**
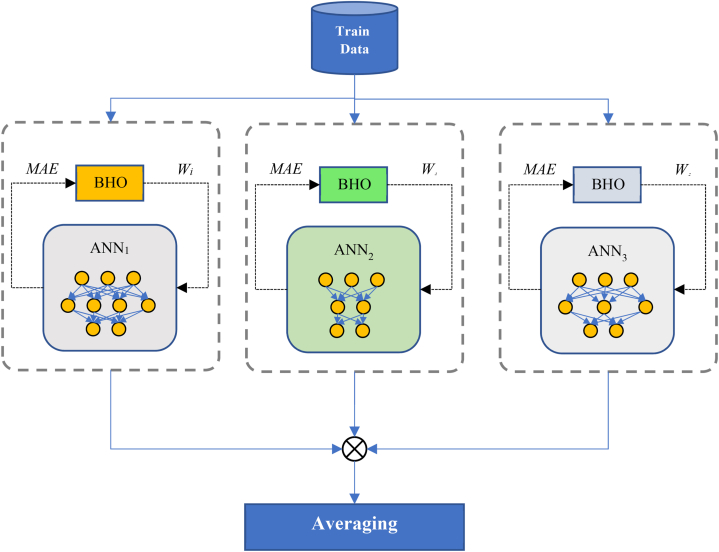
Proposed Ensemble Model based on Neural Networks and BHO Algorithm.

As mentioned, in the second step of the proposed method, a combination of ANNs and the BHO algorithm will be used to construct the ensemble model. The ensemble model used in the proposed method consists of three multi-layer perceptron networks, with the optimal topology and weight vectors for each of these networks determined using the BHO algorithm. It is worth noting that more than three multi-layer perceptron networks can be used in this ensemble model. The functioning of the proposed ensemble model is illustrated in Fig. 2.

According to the representation in Fig. 2, each ANN is independently optimized by the BHO algorithm, and during the optimization process, the optimal topology and weight vectors for the neural network are determined. Since the optimization process for each neural network is independent of the other networks, this step of the proposed method can be performed using parallel processing techniques. This significantly reduces the processing time required to construct the ensemble model.

With these explanations, in this step of the proposed method, for each ANN, a black hole optimizer will be executed, which tries to determine the best topology and weight vectors for its neural network based on the training error criterion (as its fitness function). It should be noted that the optimization steps performed by the BHO algorithm in this step of the proposed method are similar to the first step, with the difference being the use of a different fitness function and structure for the solution vectors. Therefore, this section only focuses on explaining the mentioned differences.

The solution vector in the BHO algorithm used in the proposed method determines the topology of the multi-layer perceptron network as well as the weights of the connections between neurons and their biases. Therefore, each solution vector in the optimization algorithm consists of two parts related to each other. In the first part of the solution vector, the network's topology is specified, and in the second part, the weights of neurons and biases (for the topology determined in the first part of the solution vector) are determined. Thus, the solution vectors in the optimization algorithm have a variable length determined based on the specified topology for the multi-layer perceptron network. Since the number of possible topological configurations of the multi-layer perceptron network can be infinite, constraints need to be considered for the topology part of the solution vector. Therefore, in order to limit the search space, the following constraints are considered for the first part of the solution vector.

• The number of hidden layers in each multi-layer perceptron network is set to one. Thus, the first part of the solution vector has one element, and the value in this element represents the number of neurons specified for the hidden layer of the perceptron network.
• The hidden layer of the multi-layer perceptron network has a minimum of 4 and a maximum of 15 neurons.

It should be noted that in the first part of the solution vector (topology determination part), only the number of neurons in the hidden layers is described, as the dimensions of the input and output layers of the neural network are determined based on the number of input features and the number of target classes, respectively. Since the number of neurons in the neural network is determined based on the first part of the solution vector, the length of the second part of the solution vector is also determined based on the topology specified in the first part. For a neural network with I input neurons, H hidden neurons, and P output neurons, the length of the second part of the solution vector in the BHO algorithm is equal to H×(I+1)+P×(H+1).

The fitness evaluation of each solution in the BHO algorithm is performed using a fitness function. After determining the topology and weights of the neural network by each solution vector, the network generates outputs for the training samples, which are then compared to the actual target values. The mean absolute error (MAE) is used as a measure to evaluate the performance of the neural network and the fitness of the generated solution. Therefore, the fitness function in the BHO algorithm is defined as follows [27]:

(6) $$MAE=\sum_{i=1}^{N}\left|T_{i}-Z_{i}\right|$$

In the above equation, N represents the number of training samples, T_i_ represents the target value for the i-th training sample, and Z_i_ represents the output produced by the neural network for this sample. In the proposed method, using the optimization algorithm, a structure for the neural network is determined to minimize the fitness function. The initial population in the BHO algorithm is randomly determined, and the search boundaries for the second part of the solution vector are set as [-1, +1]. Thus, each weight value for the connections between neurons and the biases of the neural network will be within this range.

**Fig. 3:**
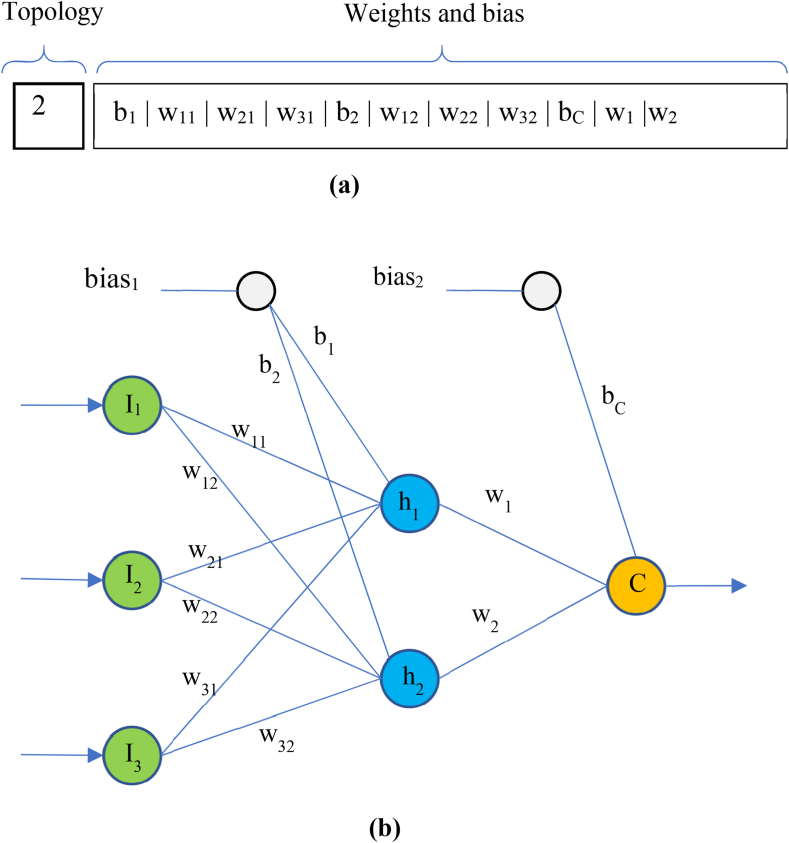
Structure of solution vectors of BHO for training ANNs (a) a sample solution vector (b) the corresponding ANN model after applying topology and weights.

Fig. 3, illustrates a sample solution vector and how it is applied on an ANN. Fig. 3a shows a solution vector. For simplicity, it is assumed that the number of input variables is 3. The first element of solution vector shows that the number of hidden neurons in this vector has been considered as 2. Therefore, the length of weight vector for training ANN will be 11. Fig. 3b shows how these weight and bias values are applied on the ANN model. After determining the topology and weight values of ANN according to the solution vector, the training samples are fed to the input layer of this model and its output is compared with the actual target values for each input sample in order to compute the fitness of the solution using equation (6).

The final step in the proposed method is estimating the training quality using the ensemble model of optimized neural networks. The proposed method utilizes the averaging technique to determine the final output of the ensemble system. The purpose of employing this technique is to reduce the error of each individual learning model by combining their outputs in a way that covers the error of each neural network through the outputs of other neural networks. Therefore, in the last step of the proposed method, each optimized neural network in the ensemble system predicts the output for the test samples, and then the average output obtained from these models is considered as the estimation result.

## 4 Implementation and results

The implementation of the proposed method for evaluating the quality of English language teaching in colleges is performed using the collected data from questionnaires and in the MATLAB 2019a software environment. In this section, after describing the process of data collection, the findings are discussed.

### 4.1 data collection

The dataset used in this study was collected through distributing questionnaires in English language classes at colleges. For this purpose, a total of 280 questionnaires were distributed, and then the accuracy of the information entered in the questionnaires was examined. In the process of data accuracy assessment, 4 questionnaires containing unanswered questions and 3 questionnaires containing invalid responses were excluded from the analysis. Therefore, the number of records in the collected database is 273. It should be noted that the target population belonged to different classes, but their educational level and teaching source were the same.

The labeling of the target variable in each database sample was performed by five experts. In this step, each evaluator determines the training quality as a ranked quantitative variable ranging from 1 to 5 by reviewing the data. Then, by calculating the average scores determined by the evaluators, the label of the target variable is determined for each data record. By doing this, the distribution of English language training quality categories for the 273 database records would be as follows: 1- Very Low (25 samples), 2- Low (40 samples), 3- Medium (79 samples), 4- Good (94 samples), and 5- Excellent (35 samples).

### 4.2 results and discussion

**Fig. 4:**
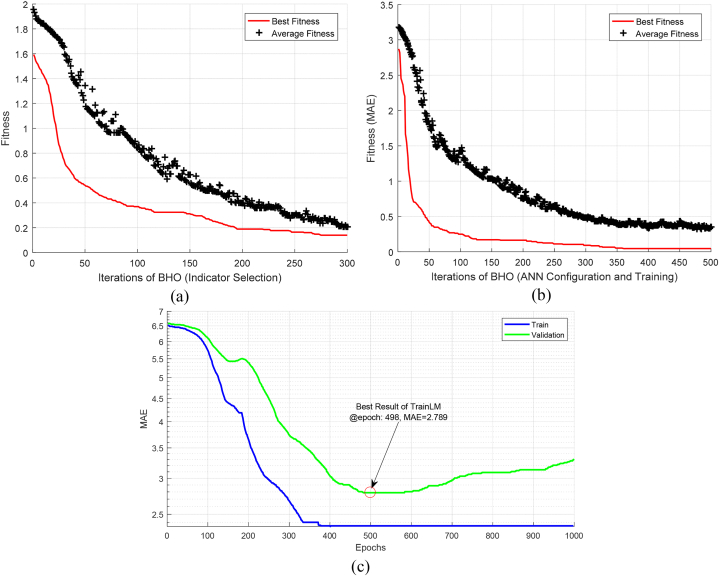
Best fitness and average fitness of the BHO algorithm in different iterations for (a) feature selection and (b) ANN model optimization phases (c) The performance of ANN during training using conventional LM training algorithm.

The proposed method was implemented using MATLAB software, and to ensure the reliability of the results, a 10-fold cross-validation technique was employed. The proposed method utilizes the BHO algorithm in two phases of feature selection and optimization of ANN models in the ensemble system. In the feature selection phase, the population size and the number of algorithm iterations were set to 140 and 300, respectively. On the other hand, for optimizing the ANN models in the proposed ensemble system, the population size was set to 200 and the number of algorithm iterations was set to 500. Fig. 4 illustrates examples of the variations in fitness of the best-discovered solutions during different iterations in these two phases. Fig. 4a and b shows that the BHO algorithm was capable of improving the discovered solution vectors for feature selection and optimizing ANN models in the ensemble system throughout different iterations. Based on the plotted graphs, the optimal solutions for feature selection and optimization of ANN models in the ensemble system were discovered at iterations 275 and 360, respectively. Additionally, the average fitness of the population tends to decrease towards minimal fitness during different iterations, indicating movement towards the global optimum.

In order to check the effectiveness of BHO algorithm in training ANN models, Fig. 4b can be compared with conventional ANN training algorithms. In Fig. 4c, the performance of ANN during training using conventional Levenberg-Marquardt (LM) training algorithm is illustrated. Fig. 4b and c are obtained based on the same training data. Fig. 4b shows that the proposed method using the BHO algorithm can reduce the training error to less than 0.03 within 360 cycles. On the other hand, Fig. 4c shows that if the ANN model is trained by the LM algorithm, the best validation error will be equal to 2.789, which is obtained after 498 cycles. These results confirm the superiority of the proposed strategy compared to the conventional LM algorithm in training ANNs. This superiority can be attributed to two factors. First, the BHO algorithm increases the probability of discovering the optimal solution by searching more efficiently in the problem space. Second, in the proposed method, the BHO algorithm tries to determine the configuration and weight vector of ANN simultaneously. However, BHO algorithm requires higher processing time compared to conventional algorithms such as LM. By running each training algorithm on a computer with an intel core i7 2.8 GHz processor and 8 GB RAM, each cycle of BHO for ANN training was executed within 1.52 s on average. This is while a training epochs in the LM algorithm were executed in 0.31 s on average. Therefore, increasing the processing time is one of the limitations of the proposed training algorithm. On the other hand, this increase in the processing time does not affect the test phase of the ANN model, and for this reason, it can be omitted.

**Fig. 5:**
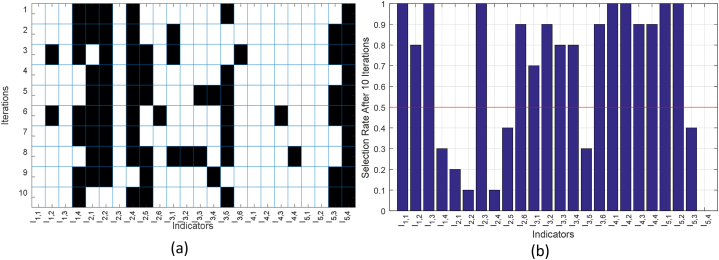
Details of the selection of prominent indicators by BHO after 10 repetitions, (a) selected indicators in each iteration, and (b) selection rate of each indicator in different iterations.

To ensure the performance of the BHO algorithm in selecting appropriate indices for evaluating the quality of teaching, the process of determining prominent indices by the BHO algorithm was repeated 10 times. In each repetition, the selected set of indices was compared with the optimal solution across all repetitions. The results of this process are presented in Fig. 5a. In this figure, each column corresponds to one of the input indicators, and each row represents one of the experiment iterations. In each row, the selected indicators are displayed in white, while the indicators eliminated by BHO are shown in black. Additionally, Fig. 5b shows the selection rate of each indicator after 10 repetitions.

By examining the plotted graphs in Fig. 5, prominent indices can be clearly identified. Fig. 5a shows that indices I_1.4_, I_2.1_, I_2.2_, I_2.4_, I_2.5_, I_3.5_, I_5.3_, and I_5.4_ were consistently disregarded in most iterations. Therefore, these indicators were deemed less important and excluded during the selection process. On the other hand, Fig. 5b indicates that 16 other indicators were selected in at least 70 % of the experimental iterations. Consequently, the proposed ANN models were trained and the assessment of English education quality was based on these 16 indicators.

To evaluate the proposed method in estimating education quality, the numerical output of the method (which is a real number in the range [1,5]) is rounded and considered as an categorical variable with values {1, 2, 3, 4, 5} for comparison with the labels determined by expert consensus. Additionally, to evaluate the effectiveness of the feature selection and model optimization techniques based on the BHO algorithm, the proposed method is compared with the following scenarios.

• All Indicators: In this scenario, the feature selection phase is ignored, and the evaluation of education quality is performed based on all input indicators. It should be noted that in this scenario, the optimization strategy of the ANN models using the BHO algorithm is still employed to improve the estimation accuracy.
• Conventional Ensemble: In this scenario, the estimation of education quality is performed based on the 16 selected indicators by the BHO algorithm through an ensemble model with basic ANNs. In this ensemble model, the training of each ANN model is conducted using the LM algorithm. In other words, instead of optimizing each ANN model by BHO algorithm, the LM algorithm was used for training them. This ensemble model includes 3 ANNs, each of which has one hidden layer. The number of neurons in hidden layer of these ANNs has been considered as: 5, 10 and 13, respectively.

**Fig. 6:**
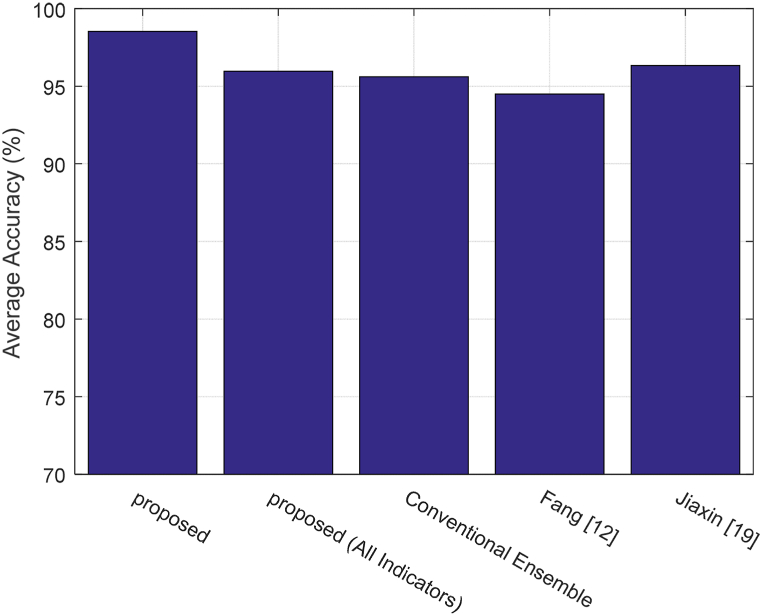
Average accuracy values of different methods in evaluating the quality of English teaching.

In addition to the above scenarios, the performance of the proposed method is compared with the methods presented by Fang [12] and Jiaxin [19]. Fig. 6 compares the average accuracy values of different methods in evaluating the quality of English teaching.

Fig. 6 shows that the proposed method performs significantly better than the compared methods with an average accuracy of 98.53 %. Comparing the accuracy of the proposed method with different scenarios demonstrates that if the feature selection process is omitted, the average estimation accuracy will be 95.97 %, indicating a decrease of 2.56 %. This indicates the positive impact of the proposed feature selection process on improving the evaluation accuracy. On the other hand, using the conventional ensemble model instead of the proposed ensemble model results in a 2.93 % decrease in accuracy. These results also confirm that the optimization strategies for the learning models can have a positive impact on improving prediction accuracy.

**Fig. 7:**
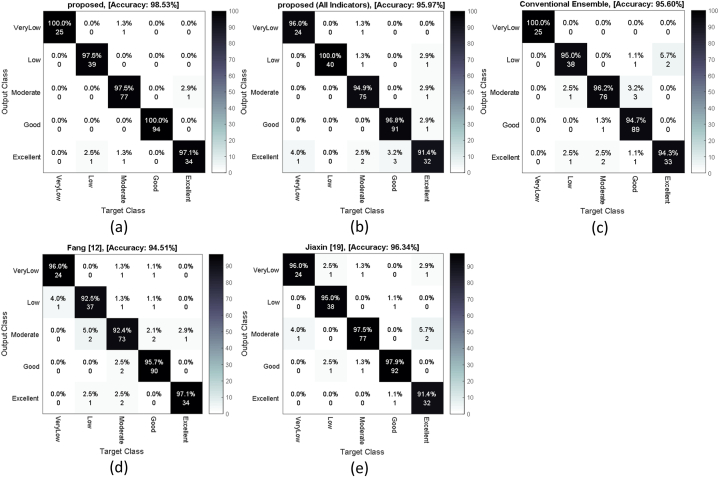
Confusion matrix of different methods for evaluating the quality of English teaching.

The obtained results demonstrate that the proposed method outperforms the Fang [12] method in all scenarios, and this improvement can be attributed to the utilization of the ensemble strategy. This is because ensemble learning can be effective in reducing the classification models' error. The closest performance to the proposed method is achieved by the Jiaxin [19] method with an average accuracy of 96.34 %. The proposed solution, by employing optimization techniques in the feature selection and ANN model configuration steps, has increased the accuracy by 2.19 % compared to this method. Fig. 7 illustrates the confusion matrix of different methods for evaluating the quality of English teaching.

Fig. 7a represents the confusion matrix of the proposed method. In the case of using all indicators for evaluation, Fig. 7b was obtained. Also, Fig. 7c demonstrates the confusion matrix of the case that the proposed classifier is replaced by the conventional ensemble. Fig. 7d and e, represent the confusion matrices of methods [12,19], respectively. In the plotted confusion matrices, rows indicate the evaluated quality by the methods for English teaching in test samples, and the matrix columns correspond to the actual class labels. The diagonal elements indicate the number of correctly classified instances. For example, the first column of the confusion matrix in Fig. 7a shows that the proposed method correctly classified all 25 instances belonging to the "Very Low" quality category. On the other hand, the values in the first row of this matrix indicate that the proposed method classified 26 instances as "Very Low" quality, out of which only one instance was misclassified and actually belonged to the "Medium" quality category. In contrast, the Jiaxin [19] method (Fig. 7e), with the closest performance to the proposed method, correctly identified 24 out of 25 instances belonging to the "Very Low" category and misclassified one instance as "Medium". Additionally, this method has three incorrect outputs in the "Very Low" category.

The interpretation of classification performance for other categories can be done in a similar manner. Further analysis reveals that the proposed method outperforms other methods in classifying instances belonging to different categories. These confusion matrices indicate that the proposed method had errors in classifying only 4 instances out of 273 instances in the database, while the Jiaxin [19] method had 10 errors. Thus, the number of erroneous instances in the proposed method is 60 % lower than the closest compared method.

For a more comprehensive evaluation of the performance of different models, precision, recall, and F-measure metrics can be used. Each of these metrics describes the classification quality for each category separately. Therefore, precision, recall, and F-measure metrics are calculated separately for each category. In this case, the target category is considered as positive, and other categories are considered as negative. These metrics can be formulated as follows [28]:

(7) $$Precision\;\left(\%\right)=\frac{TP}{TP+FP}\times 100$$

(8) $$Recall\left(\%\right)=\frac{TP}{TP+FN}\times 100$$

(9) $$F-Measure\left(\%\right)=\frac{2\times Precision\times Recall}{Precision+Recall}$$

In the above relationships, TP represents the number of correctly identified positive samples. Additionally, FP describes the number of negative samples that have been mistakenly classified as positive. Finally, FN indicates the number of positive samples that have been mistakenly classified as negative (other classes). Also, the F-measure in equation (9), calculates the harmonic average of precision and recall criteria.

**Fig. 8:**
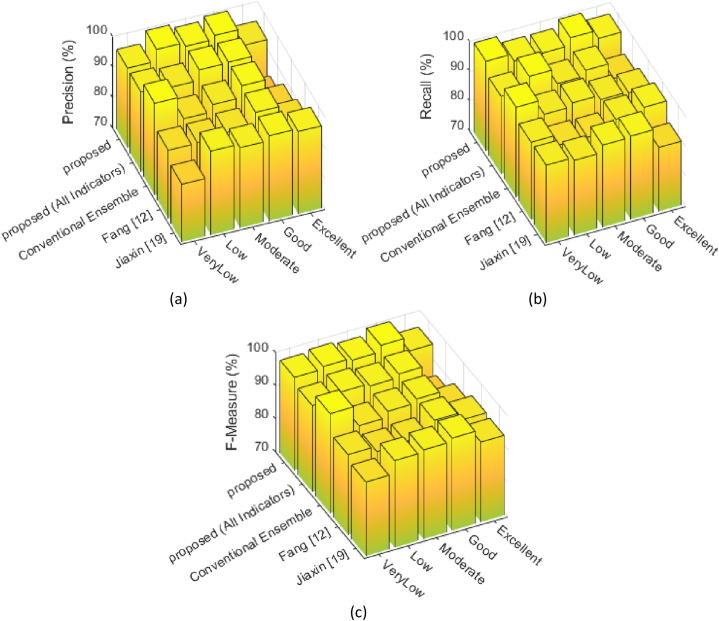
Evaluation of (a) Precision, (b) Recall, and (c) F-Measure metrics for each target class, by different methods.

In Fig. 8, the precision, recall, and F-measure plots obtained for different methods in 5 target classes are presented. In these plots, the first dimension represents the target classes, and the second dimension corresponds to the evaluation methods. These plots effectively describe the classification quality of different methods for each category.

The results presented in Fig. 8 confirm that the proposed method generally outperforms the compared methods. According to Fig. 8a, the proposed method only has lower precision for the "Excellent" class compared to the Jiaxin [19] method. However, based on Fig. 8b, the recall values of the proposed method are superior to other methods in all classes. The harmonic combination of the values of these two plots in Fig. 8c shows that the proposed method has achieved more efficient classification quality for all target classes.

Higher precision of the proposed method compared to other methods, indicate its lower false positives for different classes (according to equation (7) which increasing FP leads to decreasing precision). This means that the proposed approach can correctly identify each teaching quality level, more precisely and fewer samples have been misclassified in the target (positive) class. On the other hand, higher recall of the proposed method compared to other methods, indicate its lower false negatives for different classes (according to equation (8) which increasing FN leads to decreasing recall). This means that the proposed method could correctly identify a larger portion of actual samples belonging to each quality level. In other words, a smaller rate of samples belonging to each target class have been misclassified in other classes.

**Fig. 9:**
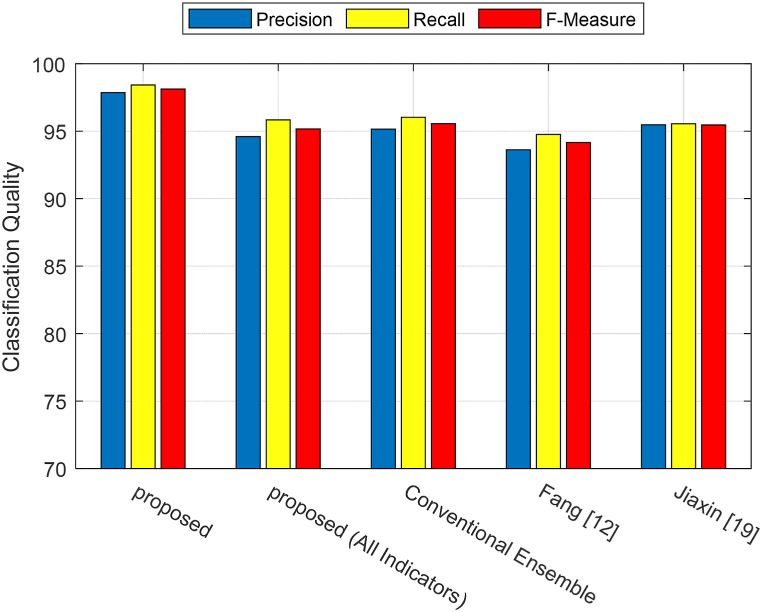
Average values of Precision, Recall, and F-Measure for different methods.

The average values of Precision, Recall, and F-Measure for different classes have been calculated, and the results are displayed in Fig. 9. According to this figure, the proposed method shows a significant improvement in terms of the average values of Precision, Recall, and F-Measure compared to the other methods. The higher precision values in the proposed method indicate that this approach has achieved better performance in accurately detecting samples from each class. In other words, the false positive rate of the proposed method is lower for different classes compared to other methods. Conversely, the higher recall values confirm that the proposed method has been able to correctly identify more samples from each target class, which results in a lower false negative rate in the classification by the proposed method. This improvement in the proposed method can be attributed to the use of the BHO algorithm in the two steps of feature selection and model optimization.

**Fig. 10:**
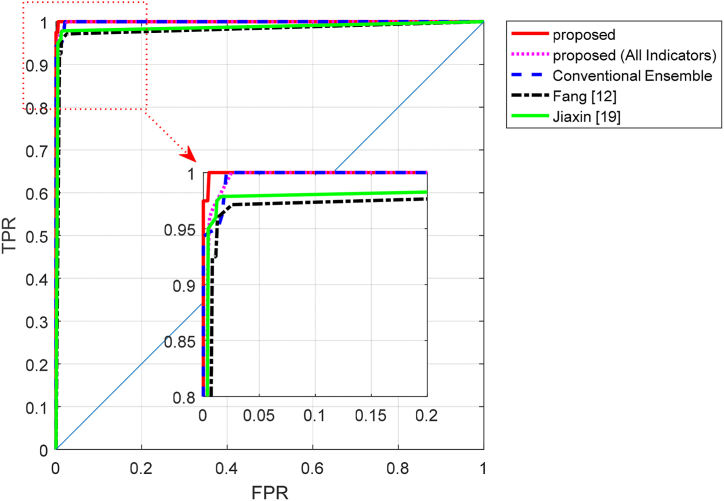
ROC curve of the proposed method compared to other methods.

Fig. 10 depicts the ROC curve of the proposed method compared to other methods. According to this figure, the proposed approach can be effective in reducing the false positive rate and increasing the true positive rate for different classes. This characteristic has led to a larger area under the ROC curve for the proposed method, indicating its superiority compared to the other methods.

The summary of the results obtained from the experiments based on accuracy, precision, recall, and F-measure metrics is provided in Table 2.

**Table 2:** Comparison of the proposed method's performance with other methods.

Method	Accuracy	F-measure	Recall	precision
Proposed	98.53	98.13	98.42	97.86
Proposed (All Indicators)	95.97	95.17	95.83	94.61
Conventional Ensemble	95.60	95.57	96.03	95.15
Fang [12]	94.51	94.17	94.76	93.62
Jiaxin [19]	96.34	95.47	95.55	95.48

Table 2 demonstrates that the proposed method outperforms other methods in terms of various metrics, allowing for a more efficient evaluation of English teaching quality. This superiority can be attributed to the techniques employed in the proposed method. The use of the BHO algorithm for selecting prominent quality evaluation indicators has ensured that redundant information and characteristics that cannot contribute to quality assessment are disregarded. This, in addition to reducing processing time, also enhances the accuracy of evaluation. Furthermore, optimizing the ANN models in the proposed ensemble system has enabled the determination of an optimal configuration for these models, ensuring their highest achievable performance. As a result, a more accurate ensemble system for evaluating the quality of English teaching can be achieved.

## 5 Conclusion

In this research, a new model for automatic evaluation of English teaching quality using ensemble learning and optimization techniques was proposed. This research introduced a new BHO-based strategy to determine prominent indicators in the evaluation of English teaching quality. This strategy selects a minimal subset of indicators relevant to the objective which can maximize the estimation accuracy based on the correlation criterion. The evaluations showed that this approach can improve the accuracy of estimating English teaching quality by at least 2.56 %. Also, in this research a new ensemble of ANN learners is used for evaluating teaching quality, where the number of neurons and weight vectors of each neural network is optimized using the BHO algorithm. In this case, the BHO algorithm attempted to create a learning model with the least training error using the MAE criterion. The findings showed that optimizing the learning models using the BHO algorithm can increase the estimation accuracy by at least 2.93 % compared to conventional training algorithms. The performance of the proposed method was evaluated using real English language training data. The results of the experiments showed that the proposed method, based on accuracy, precision, and recall metrics, can achieve values of 98.53 %, 0.9786, and 0.9842, respectively, indicating a minimum improvement of 2.19 % in accuracy compared to similar research.

Although optimizing the learning models is highly effective in improving their estimation accuracy, this strategy leads to increased computational burden in the training phase of the collective system. This can be considered a limitation of the proposed method, and future research should focus on addressing this issue. Replacing ANN models with other learning models such as decision trees, Gaussian process regression, etc., may be effective in reducing the complexity of the ensemble model. Therefore, this aspect will be investigated in future research.

## Data availability

Data will be made available on request.

## Additional information

No additional information is available for this paper.

## CRediT authorship contribution statement

**Li Guan:** Investigation, Project administration.

## Declaration of competing interest

The authors declare that they have no known competing financial interests or personal relationships that could have appeared to influence the work reported in this paper.
